# Complications after radical gastrectomy following FOLFOX7 neoadjuvant chemotherapy for gastric cancer

**DOI:** 10.1186/1477-7819-9-110

**Published:** 2011-09-26

**Authors:** Zi-Yu Li, Fei Shan, Lian-Hai Zhang, Zhao-De Bu, Ai-Wen Wu, Xiao-Jiang Wu, Xiang-Long Zong, Qi Wu, Hui Ren, Jia-Fu Ji

**Affiliations:** 1 Department of Surgery, Key Laboratory of Carcinogenesis and Translational Research (Ministry of Education), Peking University School of Oncology, Beijing Cancer Hospital & Institute, Beijing 100142, China

**Keywords:** Gastric cancer, neoadjuvant chemotherapy, complication, FOLFOX7, surgery

## Abstract

**Background:**

This study assessed the postoperative morbidity and mortality occurring in the first 30 days after radical gastrectomy by comparing gastric cancer patients who did or did not receive the FOLFOX7 regimen of neoadjuvant chemotherapy.

**Methods:**

We completed a retrospective analysis of 377 patients after their radical gastrectomies were performed in our department between 2005 and 2009. Two groups of patients were studied: the SURG group received surgical treatment immediately after diagnosis; the NACT underwent surgery after 2-6 cycles of neoadjuvant chemotherapy.

**Results:**

There were 267 patients in the SURG group and 110 patients in the NACT group. The NACT group had more proximal tumours (P = 0.000), more total/proximal gastrectomies (P = 0.000) and longer operative time (P = 0.005) than the SURG group. Morbidity was 10.0% in the NACT patients and 17.2% in the SURG patients (P = 0.075). There were two cases of postoperative death, both in the SURG group (P = 1.000). No changes in complications or mortality rate were observed between the SURG and NACT groups.

**Conclusion:**

The FOLFOX7 neoadjuvant chemotherapy is not associated with increased postoperative morbidity, indicating that the FOLFOX7 neoadjuvant chemotherapy is a safe choice for the treatment of local advanced gastric cancer.

## Background

Long-term survival is the gold standard in the assessment of gastric cancer. The complete surgical resection of tumours with negative margins (R0 resection) has been considered the most effective treatment for gastric cancer and is associated with improved long-term survival [1,2]. The concept of neoadjuvant chemotherapy has recently been widely accepted to increase the R0 resection rate and the long-term survival in patients with gastric cancer. To date, owing to the results of the Medical Research Council Adjuvant Gastric Infusional Chemotherapy (MAGIC) trial, perioperative chemotherapy for locally advanced resectable gastric cancer has become a grade A recommendation [3]. Although the role of neoadjuvant therapy has now been established, the optimal regimen remains to be determined. Various regimens of neoadjuvant chemotherapy in gastric cancer have been shown to induce tumour responses [4]. But the potential accompanied disadvantages, including increased surgical complications, cannot be ignored. In addition, patients who may not be eligible to receive postoperative adjuvant therapy because of poor performance status secondary to postoperative complications may benefit from receiving systemic therapy first. There are limited data available regarding postoperative morbidity and mortality in patients receiving neoadjuvant chemotherapy for gastric cancer. If neoadjuvant chemotherapy is to be considered as a therapeutic option in patients with locally advanced gastric cancer, it is necessary to verify that treatment can be delivered safely without an increase in postoperative morbidity and mortality.

Neoadjuvant chemotherapy with the FOLFOX regimen for local advanced gastric cancer has been performed for years in our centre. The current retrospective study was undertaken to assess the postoperative morbidity and mortality in patients receiving FOLFOX7 neoadjuvant chemotherapy prior to radical gastrectomy for local advanced gastric cancer in comparison to patients who underwent gastrectomy alone during the same time period, at the same institutions, by the same surgeons.

## Methods

Patients' medical records and histologic data during the period from April 18, 2005 to October 20, 2009 were retrospectively studied. Patients included in the study had histologically confirmed gastric adenocarcinomas and received curative gastrectomy with D2 lymph node dissection by the same surgeons at the department of Surgery of the Beijing Cancer Hospital & Institute and the Peking University School of Oncology. Of these, there were 267 patients (SURG group) who received surgical treatment immediately after diagnosis and another 110 patients (NACT group) who first received FOLFOX7 neoadjuvant chemotherapy. Information regarding postoperative morbidity and mortality was available for each patient studied. Mortality was defined as a lethal outcome during the operation or within the first 30 postoperative days. Complications were also considered if they occurred in the same period. All patients were diagnosed prior to therapy with resectable local advanced gastric cancer as T3-4 N any and M0, according to the 1997 American Joint Committee on Cancer criteria (AJCC). All patients routinely underwent chest and abdominal CT and laparoscopy for staging purposes and must have had measurable disease to enable response monitoring. Endoscopic ultrasound (EUS) was also performed for patients in the neoadjuvant arm of the study. Patients were allocated to either of the treatment arms based on patient preference after the pros and con of each treatment modality were fully explained using a standard pro forma. Patients who required urgent surgery for obstruction, perforation, or bleeding and patients who did not receive radical gastrectomy were not included in this study. Induction chemotherapy with 2 to 6 cycles of FOLFOX7 was completed on an outpatient basis, which consisted of a 2-hour infusion of folinic acid at 400 mg/m^2 ^followed by a 5-FU 46-hour infusion of 2,400 mg/m^2 ^every 2 weeks. Oxaliplatin at 130 mg/m^2 ^was infused for 2 hours on day 1. Anti-emetics were routinely prescribed, and granulocyte colony stimulating factor (G-CSF) was regularly used. Surgery was performed 2-8 weeks after completion of neoadjuvant therapy, and gastric resection was completed in a similar fashion for both groups. Patients received either an en bloc radical proximal, distal, or total gastrectomy depending on the anatomic location of the cancer with a view to R0 resection. A D2 lymphadenectomy was performed according to the Japanese Research Society for Gastric Cancer guidelines [5]. Intra-operative frozen sections were used liberally for confirmation of negative margins. All patients received the same perioperative management such as prophylactic antibiotics, nutritional support (total parenteral nutrition, TPN), and drainage. Nasogastric tubes were not routinely used unless there were signs of obstruction.

Demographic, clinical, and pathologic characteristics of the two groups were analysed. Statistical analysis was performed with the SPSS 13.0 statistical software. The comparisons among groups were performed by Student's t test and the chi-square test. P values are reported for a two-tailed test with P < 0.05 considered significant.

## Results

### Patient demographics and Clinical characteristics

Patient demographics and clinical characteristics are outlined in Table 1. The group included 277 men and 100 women. The median age was 59 years. NACT patients tended to be younger than SURG patients (56 years vs. 60 years; P = 0.008). NACT patients were more likely to have proximal tumours with 46% being located at the gastroesophageal junction/cardia compared to 24% in the SURG group. Conversely, SURG patients were more likely to have distal lesions (P = 0.000) as reflected by the surgeries performed with 33% of NACT patients undergoing distal subtotal gastrectomy compared with 58% of SURG patients (P = 0.000). As previously mentioned, all patients had locally advanced cancers defined as T3 or T4 with or without nodal involvement as determined by physical examination, imaging and endoscopy. Although clinical staging before treatment was similar in the two groups, pathologic staging (according to the AJCC system) showed less cases of the T (P = 0.000) and N (P = 0.009) stages in the NACT group as compared with the SURG group, which is consistent with a tumour downstaging effect. More than 50% of patients in the NACT group acquired major response and nearly 30% of patients got experienced tumour downstaging in the T stage.

**Table 1:** Patient demographics and clinical characteristics

	NACT (%) n = 110	SURG (%) n = 267	P value
**Age, years**	55.5 ± 11.5	59.7 ± 12.2	0.008
Median (range)	56.0(26-82)	59(25-85)	
**Gender**			0.576
Male	83(75.5%)	194(72.7%)	
Female	27(24.5%)	73(27.3%)	
**Tumor location**			0.000
Proximal	51 (46.4%)	64 (24.0%)	
Body	17(15.5%)	40(15.0%)	
Distal	41(37.3%)	157(58.8%)	
Gastric remnant	0(0%)	2(0.7%)	
Other(total)	1(0.9%)	4(1.5%)	
**Pre-treatment clinical T Staging/Pathological T Staging**			
-T0	0(0%)/6(5.5%)	0(0%)/0(0%)	0.000
-T1	0(0%)/6(5.5%)	0(0%)/1(0.4%)	
-T2	0(0%)/13(11.8%)	0(0%)/28(10.5%)	
-T3	90(81.8%)/73(66.4%)	251(94%)/221(82.8%)	
-T4	20(18.2%)/12(10.9%)	16(6.0%)/17(6.4%)	
**Pathological N Staging**			0.009
-N0	34(30.9%)	44(16.5%)	
-N1	47(42.7%)	119(44.6%)	
-N2	17(15.5%)	65(24.3%)	
-N3	12(10.9%)	39(14.6%)	

NS = not significant

### Preoperative status

The performance status of all patients according to the Eastern Cooperative Oncology Group was either 0 or 1. Twenty-four (6%) of all 367 patients had BMI values greater than 28. There were no significant differences in BMI values between the NACT and SURG patients (P = 0.773). The mean preoperative serum albumin was 42.77 g/L in the NACT group and 42.15 g/L in the SURG group (P = 0.211). There was also no difference in the preoperative CEA, CA199 or haemoglobin between the two groups. The white blood cell counts of both groups were within the normal range, although counts were lower in the NACT group than in the SURG group (P = 0.000), which is also consistent with a chemotherapy effect. SURG patients were more likely to have a comorbid illness (P = 0.033). Cardiovascular disease with a history of previous myocardial infarction, ischemic heart disease, and hypertension requiring treatment was prevalent in 16% of NACT patients and 22% of SURG patients (P = 0.083). There were no significant differences in the prevalence of diabetes mellitus, pulmonary, renal, or liver diseases, or surgical histories between the NACT and SURG patients (Table 2).

**Table 2:** Patient preoperative status

	NACT (%) n = 110	SURG (%) n = 267	P value
**Body mass index (BMI)**	22.92 ± 3.12	23.03 ± 3.56	0.773
**White blood cells**	5.29 ± 1.84	6.25 ± 2.10	0.000
**Hemoglobin**	121.72 ± 21.92	123.93 ± 28.26	0.417
**Serum albumin**	42.83 ± 4.25	42.18 ± 4.62	0.211
**CEA**	15.986 ± 67.377	4.357 ± 9.192	0.081
**CA199**	175.836 ± 803.631	81.270 ± 394.942	0.255
**Comorbid illness**	44(40.0%)	139(52.1%)	0.033
Cardiovascular	20(18.2%)	71(26.6%)	0.083
Pulmonary	3(2.7%)	9(3.4%)	1.000
Gastric Disease	1(0.9%)	12(4.5%)	0.119
Renal	0(0.0%)	5(1.9%)	0.327
Diabetes mellitus	6(5.5%)	28(10.5%)	0.121
Liver disease	3(2.7%)	10(3.7%)	0.764
Operation history	20(18.2%)	48(18.0%)	0.963
Tuberculosis history	1(0.9%)	3(1.1%)	1.000
Others	7(6.4%)	14(5.2%)	0.666

A total of 410 cycles of preoperative chemotherapy were delivered to NACT patients, with a median of four cycles per patient (ranging from two to six cycles per patient). Two patients (2%), 14 patients (13%), 5 patients (5%), 84 patients (76%), 1 patient (1%) and 4 patients (4%) received one, two, three, four, five or six cycles, respectively, of chemotherapy before surgery. No dose reduction was required in the 410 cycles delivered, and there were no significant differences in the presence of complications among the patients receiving different numbers of chemotherapy cycles.

### Operative parameters

Mean total operative time (excluding anaesthetic preparation and repositioning of the patient) was 200 minutes in the NACT group and 183 minutes in the SURG group (P = 0.005). Consequences of chemotherapy, such as tissue oedema, may require increased surgical time for careful dissection. Mean operative blood loss was 235 mL in the NACT group and 197 mL in the SURG group (P = 0.061). Perioperative transfusion was completed in 10% of NACT patients and 17% of SURG patients (P = 0.063), including those procedures only for the correction of preoperative anaemia. The NACT patients had more total/proximal gastrectomies than the SURG group (P = 0.000). There were no significant differences between the two groups in the extent of resection, multi-visceral resection, type reconstruction or number of nodes harvested (Table 3). Multi-visceral resection, including cholecystectomy, splenectomy, partial pancreatectomy, partial colectomy and partial liver resection, was performed in 9.7% of SURG patients as compared to 14.5% of NACT patients (P = 0.177).

**Table 3:** Patient operative parameters

	NACT (%) n = 110	SURG (%) n = 267	P value
**Mean total operative time**	200.4 ± 56.6 mins	182.8 ± 53.5 mins	0.005
**Mean operative blood loss**	235.1 ± 185.3 ml	197.5 ± 149.9 ml	0.061
**Patients with transfusion**	10 (9.1%)	44(16.5%)	0.063
**Type of resection**			0.000
Total gastrectomy (via abdomen)	45(40.9%)	66(24.7%)	
Total gastrectomy (via abd & cht)	2(1.8%)	0(0%)	
Distal gastrectomy	36(32.7%)	156(58.4%)	
Proximal gastrectomy (via abd)	24(21.8%)	45(16.9%)	
Proximal gastrectomy (via abd & cht)	3(2.7%)	0(0.0%)	
**Radical resection**			0.112
R0	110(100.0%)	260(97.4%)	
R1 or R2	0(0%)	7(2.6%)	
**Multivisceral resection**	16(14.5%)	26(9.7%)	0.177
**Reconstruction**			
Total gastrectomy	47(42.7%)	66(24.7%)	1.000
Roux-en-Y	4(8.5%)	6(9.1%)	
Jejunal interposition with a ρ-pouch	43(91.5%)	60(90.9%)	
Distal gastrectomy Billroth-I	36(32.7%) 35(97.2%)	156(58.4%) 123(78.8%)	0.074
Billroth-II	1(2.8%)	17(10.9%)	
Roux-en-Y	0(0.0%)	15(9.6%)	
Jejunal interposition with a ρ-pouch	0(0.0%)	1(0.6%)	
Proximal gastrectomy	27(24.5%)	45(16.9%)	0.238
Esophagogastric anastomosis	26(96.3%)	43(95.6%)	
Jejunal interposition with a ρ-pouch	0(0.0%)	2(4.4%)	
Others	1(3.7%)	0(0.0%)	
manual anastomosis	3(2.9%)	1(0.4%)	0.072
**Median no. of nodes harvested**	32.4 ± 14.0	32.3 ± 13.5	0.963

abd: abdomen; cht: chest

### Complications

Complications occurred in 57 of the 377 patients undergoing resection and were not significantly different between the two groups (P = 0.075, Table 4). The overall median postoperative hospital stay was 11 days in the NACT group and 13 days in the SURG group (P = 0.015). For patients with no complications, the median postoperative stay was 10 days in both groups (P = 0.952), and for those suffering morbidity, median values were 17 days in the NACT group and 24 days in the SURG group (P = 0.174).

**Table 4:** Morbidity and mortality

	NACT (%) n = 110	SURG (%) n = 267	P value
**Patients with complications**	11(10.0%)	46(17.2%)	0.075
**Postoperative LOS (days)**	11.0 ± 4.7(5-40)	13 ± 8.9(6-79)	0.015
**Postoperative LOS with complications (days)**	16.8 ± 10.9(7-40)	24.0 ± 16.4(6-79)	0.174
**Postoperative LOS without complications (days)**	10.4 ± 2.8(5-19)	10.4 ± 3.1(6-27)	0.952
**Nonsurgical complications**			
Pneumonia	1(0.9%)	4(1.5%)	1.000
Pleural effusion	2(1.8%)	2(0.7%)	0.583
gastric motility disorder	2(1.8%)	10(3.7%)	0.521
Mental status changes	0(0.0%)	1(0.4%)	1.000
Others (Diarrhea, Hiccup,	0(0.0%)	4(1.5%)	0.326
Thrombocytopenia) No. of patients	4(3.6%)	21(7.9%)	0.134
**Surgical complications**			
Anastomotic leak	2(1.8%)	9(3.4%)	0.520
Intra-abdominal abscess	3(2.7%)	8(3.0%)	1.000
Postoperative bowel obstruction/ileus	2(1.8%)	3(1.1%)	0.631
Postoperative hemorrhage	0(0.0%)	4(1.5%)	0.326
Wound infection	0(0.0%)	2(0.7%)	1.000
No. of patients	7(6.4%)	26(9.7%)	0.292
**Reexploration**	1(0.9%)	9(3.4%)	0.292
**Mortality**	0(0.0%)	2(0.7%)	1.000

LOS = length of stay; NS = not significant

Overall, nonsurgical complications and surgical complications were similar between the NACT and SURG groups. The most common nonsurgical complications were gastric motility disorder and pulmonary problems. Anastomotic leak and intra-abdominal abscess were the most common surgical complications in these patients. Of the 377 patients undergoing radical gastrectomy, there were two deaths (both in the SURG group, 0.7%), and ten patients (one in the NACT group) required early reoperation. Neoadjuvant chemotherapy did not increase the risk of postoperative complications, mortality, or the need for reoperation. The two deaths in the SURG group were the result of multi-organ failure on day 45 following oesophago-gastric anastomotic leak, which underwent late re-exploration, and septic complications on postoperative day 8 related to the abdominal abscess, respectively. Nine SURG patients underwent re-exploration. Six were for postoperative leak with one eventual death, two for postoperative haemorrhage and one for abdominal abscess. By Multinomial Logistic analysis, there was no significant association between the development of complications and the following variables: age, sex, tumour location, type of resection, extent of resection (R0), multi-visceral resection, nodal dissection, pathologic AJCC stage, and whether the patient received neoadjuvant chemotherapy.

## Discussion

The goal of surgery for gastric carcinoma is a curative resection that involves the removal of all gross cancer and regional lymph nodes without leaving any macroscopically visible cancer lesions. Neoadjuvant chemotherapy for gastric cancer aims to downstage the tumour, thus improving the curative resectability of locally advanced tumours and eventually increasing the survival of patients. Since the publication of the results from the MAGIC trial, substantial scientific evidence has suggested the benefits of perioperative (preoperative and postoperative) chemotherapy for locally advanced gastric cancer [3]. Up to this point, many neoadjuvant chemotherapy treatments for gastric cancer have been used with varying success to downstage locally advanced gastric cancers [4], and finding a better regimen of choice for neoadjuvant chemotherapy is undoubtedly the focus of this area. However, there are limited data available regarding postoperative morbidity and mortality in patients receiving neoadjuvant chemotherapy of different regimens for gastric cancer, and most studies providing detailed analysis of postoperative complications in patients receiving neoadjuvant chemotherapy have not included a comparative group of patients undergoing surgery alone [6-8]. It is necessary to assess the influence of preoperative chemotherapy on surgery if it is to be considered as a standard treatment, especially with the increasing number of new drugs available for clinical application. Clinical trials concerning neoadjuvant therapy with the FOLFOX regimen for local advanced gastric cancer have been performed in our department since 2002. This retrospective study aimed to examine postoperative morbidity and mortality in patients receiving neoadjuvant FOLFOX7 chemotherapy compared to a group of patients undergoing surgical resection only during the same time frame and by the same surgeons. The results indicated that Oxaliplatin-based neoadjuvant chemotherapy does not increase the risk of postoperative complications in patients undergoing gastrectomy with D2 lymphadenectomy for gastric cancer.

Surgical morbidity and mortality following gastrectomy can be substantial. The most frequent complications following gastrectomy for gastric cancer are pulmonary problems, anastomotic leakage, intra-abdominal abscess, and wound infection[9-12]. Factors reported to influence morbidity in patients undergoing gastrectomy for gastric cancer include multi-organ resection, especially splenectomy and distal pancreatectomy, age greater than 70 years with underlying cardiopulmonary or renal disease, and extended lymph node dissection. In patients with gastric cancer receiving neoadjuvant chemotherapy followed by resection, postoperative morbidity ranges from 23% to 40% and mortality from 0% to 10% [6-8,13-17]. These figures are similar to reports of morbidity and mortality in patients undergoing gastric resection without neoadjuvant chemotherapy [9-12,18-23] and are similar to findings in our study, which also support the observation that neoadjuvant chemotherapy does not increase morbidity and mortality. In the current study, morbidity was 10.9% in the NACT patients and 17.2% in the SURG patients. There were two postoperative deaths, both in the SURG group (P = 1.000). No significant factors were found to be associated with the development of complications.

Only 24 (6%) patients with BMI values greater than 28 were included in our study, reflecting the differences between patients populations in the East and West and potentially explaining the lower incidence of morbidity and mortality in the Eastern study. Although the SURG patients were older (P = 0.008), increasing age was not associated with the development of postoperative complications and may be associated with the longer postoperative hospital stay (P = 0.015). Both groups of patients had similar pre-treatment cancer stages. We believe that the preponderance of lower numbers of T and N stages in the NACT group as opposed to those in the SURG group are the result of the downstaging effect following neoadjuvant chemotherapy.

Although D2 lymphadenectomy was routinely performed in our patients, multi-visceral resection, especially distal pancreatectomy and splenectomy, was rarely necessary. Others have reported increasing age and extended lymphadenopathy with multi-visceral resection to be associated with increasing mortality [10,12,21]. We agree that extended lymphadenectomy combined with multi-visceral resection, specifically splenectomy with or without distal pancreatectomy, should be avoided unless there is direct extension of the tumour mandating resection to achieve negative margins [20,24]. The low rate of major surgical complications and mortality secondary to surgical complications in the current cohort may be partially related to our limited use of multi-visceral resection.

Re-laparotomy for complications of gastrectomy is necessary in 2% to 12% of cases [9,12,19-21]. In a large series of 700 gastrectomies reported by Shchepotin *et al.*, 40 patients (5.7%) underwent reoperation with an associated mortality of 62.5%. Anastomotic leakage and pancreatic necrosis were the most common indications for reoperation. Ten patients in the current series, including six patients with leak, underwent re-exploration. Postoperative pancreatitis was not observed in our series. With improved surgical techniques, anastomotic leaks appear to be decreasing in incidence. We agree with the opinion that leaks should be managed conservatively and reoperation reserved for patients in whom conservative management is unsuccessful [23]. In the current series, two patients in the SURG group who underwent reoperation died. Operative mortality rates following gastrectomy range from 0% to 10% [6-8,13-17]. Our overall postoperative mortality of 0.7% is within this range, and FOLFOX7 neoadjuvant chemotherapy was not associated with an increase in mortality, which is consistent with results in other series in which various regimens of neoadjuvant chemotherapy were used [6,8,14,15,25].

## Conclusions

One of the theoretical advantages of neoadjuvant therapy is the enhanced ability to deliver multimodality therapy to all suitable patients and not delay a patient's therapy because of a prolonged recovery from surgery or inadequate resection. In summary, we have shown that neoadjuvant chemotherapy with FOLFOX7 can be delivered without increasing surgical morbidity and mortality compared to gastrectomy alone. In this respect, neoadjuvant chemotherapy with FOLFOX7 is a safe candidate for the treatment of local advanced gastric cancer. However, this is a retrospective study from a single centre, and future studies are needed to confirm these results. In China, a randomised multicentre phase III study conducted by our centre is underway to evaluate the effectiveness of neoadjuvant chemotherapy with the FOLFOX regimen for locally advanced gastric cancer. Further investigation is warranted to determine the most efficacious and least toxic combination regimen of neoadjuvant/adjuvant therapies for treating gastric cancer.

## Competing interests

The authors declare that they have no competing interests.

## Authors' contributions

JFJ was the lead author and surgeon for all of the patients. ZYL undertook the literature research. ZYL, FS, LHZ and HR gathered information on the patients and contributed to writing of the paper. ZDB, AWW, XJW and XLZ were the co-surgeon on the cases. ZYL, QW and FS performed the data and statistical analysis. ZYL prepared the manuscript. All authors read and approved the final manuscript.

## Consent

Written informed consent was obtained from the patient for publication of this case report and any accompanying images. A copy of the written consent is available for review by the Editor-in-Chief of this jounal.
